# Risk Factors for Acquisition of Fluoroquinolone or Aminoglycoside Resistance in Addition to Carbapenem Resistance in *Pseudomonas aeruginosa*

**DOI:** 10.2174/1874285801812010321

**Published:** 2018-09-28

**Authors:** Kosuke Kosai, Norihito Kaku, Naoki Uno, Tomomi Saijo, Yoshitomo Morinaga, Yoshifumi Imamura, Hiroo Hasegawa, Taiga Miyazaki, Koichi Izumikawa, Hiroshi Mukae, Katsunori Yanagihara

**Affiliations:** 1 Department of Laboratory Medicine, Nagasaki University Hospital, Nagasaki, Japan; 2 Department of Laboratory Medicine, Nagasaki University Graduate School of Biomedical Sciences, Nagasaki, Japan; 3 Department of Respiratory Medicine, Nagasaki University Graduate School of Biomedical Sciences, Nagasaki, Japan; 4 Department of Infectious Diseases, Nagasaki University Graduate School of Biomedical Sciences, Nagasaki, Japan

**Risk Factors for Acquisition of Fluoroquinolone or Aminoglycoside Resistance in Addition to Carbapenem Resistance in *Pseudomonas Aeruginosa***

The Open Microbiology Journal, **2017**, 11-92

The correct Table 2 is mentioned below:

**Table 2:** Univariate and multivariate analyses of risk factors for acquisition of fluoroquinolone or aminoglycoside resistance in addition to imipenem resistance in *Pseudomonas aeruginosa*.

	Univariate analysis			Multivariate analysis		
	OR	95%CI	P	OR	95%CI	P
Resistance to FQs						
Transplantation	1.74	0.80-3.78	0.161			
Use of corticosteroids (≥5 mg/day) or other immunosuppressive agents	1.81	0.93-3.52	0.082			
Surgery	0.65	0.35-1.21	0.173			
Exposure to first-generation cephalosporins	0.60	0.30-1.19	0.143			
Exposure to second-generation cephalosporins	0.28	0.08-1.01	0.051			
Exposure to FQs	5.30	2.52-11.14	<0.001	5.73	2.67-12.30	<0.001
Exposure to AGs	3.92	1.30-11.87	0.016			
MBL production	6.67	1.78-24.91	0.005	7.90	2.01-31.04	0.003
Resistance to AGs						
Use of anticancer drugs	2.57	0.64-10.40	0.184			
Use of urinary catheter	0.42	0.13-1.38	0.152			
Exposure to antipseudomonal penicillins	0.24	0.05-1.14	0.073	0.16	0.03-0.89	0.036
Exposure to AGs	6.04	1.59-23.00	0.008	9.00	1.92-42.19	0.005
MBL production	7.35	1.89-28.65	0.004	8.49	1.87-38.52	0.006

OR, odds ratio; CI, confidence interval; FQs, fluoroquinolones; AGs, aminoglycosides; MBL, metallo-β-lactamase

The original table provided was:

**Table 2:** Proportion of resistant and MBL-producing strains by presence of FQ or AG resistance in imipenem-resistant *Pseudomonas aeruginosa*.

		**Resistance to FQs**			**Resistance to AGs**		
	**All strains** **(n = 169)**	**Yes** **(n = 66)**	**No** **(n = 103)**	**P**	**Yes** **(n = 12)**	**No** **(n= 157)**	**P**
Resistance to antibiotics							
Piperacillin^1)^	36 (21.3)	21 (31.8)	15 (14.6)	0.003	4 (33.3)	32 (20.4)	0.347
Ceftazidime^1)^	53 (31.4)	33 (50.0)	20 (19.4)	<0.001	8 (66.7)	45 (28.7)	0.022
Cefepim	38 (22.5)	28 (42.4)	10 (9.7)	<0.001	7 (58.3)	31 (19.7)	0.006
Aztreonam	91 (53.8)	45 (68.2)	46 (44.7)	0.004	9 (75.0)	82 (52.2)	0.146
Ciprofloxacin^1)^	49 (29.0)	-	-	-	8 (66.7)	41 (26.1)	0.011
Levofloxacin	60 (35.5)	-	-	-	6 (50.0)	54 (34.4)	0.350
Gentamicin^1)^	12 (7.1)	8 (12.1)	4 (3.9)	0.042	-	-	-
Amikacin^1)^	4 (2.4)	3 (4.5)	1 (1.0)	0.274	-	-	-
MBL production	14 (8.3)	11 (16.7)	3 (2.9)	0.003	4 (33.3)	10 (6.4)	0.010

Values are expressed as the number (%). FQs, fluoroquinolones; AGs, aminoglycosides; MBL, metallo-β-lactamase ^1)^Minimum inhibitory concentrations (MICs) for piperacillin, ceftazidime, ciprofloxacin, gentamicin and amikacin were not measured in eleven, one, one, one, and thirteen strains, respectively.

